# Can School-Based Physical Activity Projects Such as Skipping Hearts Have a Long-Term Impact on Health and Health Behavior?

**DOI:** 10.3389/fpubh.2020.00352

**Published:** 2020-08-14

**Authors:** Lisa Baumgartner, Tanja Postler, Christine Graf, Nina Ferrari, Bernhard Haller, Renate Oberhoffer-Fritz, Thorsten Schulz

**Affiliations:** ^1^TUM Department of Sport and Health Sciences, Institute of Preventive Pediatrics, Technical University of Munich, Munich, Germany; ^2^Institute of Movement and Neurosciences, German Sport University Cologne, Cologne, Germany; ^3^Institute of Medical Informatics, Statistics and Epidemiology, Technical University of Munich, Munich, Germany

**Keywords:** physical activity, health behavior, accelerometry, body composition, long-term evaluation, children, adolescents

## Abstract

Low physical activity, limited motor skills, and an increased number of overweight or obese children are major public health problems. Numerous school-based programs try to improve physical activity and health behavior in children but investigations on sustainable effects of these programs are rare. Therefore, we examined the long-term effects of the *Skipping Hearts* health promotion project. 486 children (57.7% female, 9.0 ± 0.6 years at baseline) participated in this non-randomized controlled longitudinal trial within a follow-up period of 3.5 years. Of these, 286 subjects received a one-time 90-min workshop in rope skipping (Basic-Workshop) and 140 additionally received 10 lessons in rope skipping (Champion-Program), 78 students served as controls. Anthropometrics, blood pressure, motor skills, screen-based media use, self-assessment of physical fitness, and physical activity were collected at both measurement points; endurance capacity and health-related quality of life only at follow-up. Standard deviation scores of body-mass-index (η^2^ = 0.005) and systolic blood pressure (η^2^ = 0.006) decreased, while diastolic blood pressure *(*η^2^ = 0.004), motor performance (η^2^ < 0.001), physical fitness, subjective physical activity (η^2^ = 0.008), and screen-based media use (η^2^ = 0.001) increased without significant difference in development between groups (all *p* > 0.05). At follow-up, groups did not differ in endurance capacity (η^2^ = 0.010) and health-related quality of life (η^2^ < 0.001). *Skipping Hearts* does not affect the long-term improvement of health status, motor performance, or health behavior. To improve the effects, the project should be implemented as a daily routine in schools to force the transfer of health behavior-related knowledge. Nevertheless, the project offers a physical activity that can be performed in children's everyday life without high costs.

## Introduction

Poor physical activity in childhood and adolescence is associated with a higher risk for cardiovascular and chronic diseases and is, therefore, a major public health problem (1, 2). Several studies have found an adverse correlation between regular physical activity in childhood and adolescence and body mass index (BMI), waist circumference, and blood pressure (3–6). Having these risk factors at an early age leads to higher mortality and morbidity in adulthood (7). Ward et al. (8) report a 1 in 10 probability that 5-year-old obese children will not be obese by the age of 35 years.

Although countless projects promoting physical activity in this span of life exist, only 26% of German children and adolescents meet the recommendation of at least 60 min of moderate-to-vigorous physical activity (MVPA) per day (9, 10). It is of great significance that the physical activity level declines with increasing age during childhood and adolescence. Jekauc et al. (11) found an age-related continuous decrease in physical activity, especially in girls. The probability of meeting the World Health Organization's physical activity recommendations decreases every year by 17% in childhood and by 19% in adolescence (11).

The maturation of distinct motor skills is of crucial importance for early childhood development. Several studies have shown a positive correlation between motor skills and total, light-to-moderate, and MVPA in childhood and adolescence (12–14). Regular physical activity is not only important for physical health, but it is also associated with psychical well-being at an early age (15). Finne et al. (16) have shown a positive relationship between physical activity and health-related quality of life (HRQoL) and a negative association between HRQoL and screen-based media use (SBM), respectively. Therefore, the need for programs promoting health behavior, health status, and physical performance in childhood is particularly high.

Numerous projects and intervention studies, both national and international, promote physical activity in this age group with the main goal of changing their behavior towards a healthier lifestyle. The conceptions of these projects range from one-time offers to regular interventions lasting several hours and weeks, from projects limited to physical activity promotion to multimodal projects including nutrition or stress management, and from the integration only of children to the additional inclusion of parents (17). School seems to be particularly suitable as a setting for such projects since nowhere else can so many different children and adolescents be reached at the same time (18). Furthermore, Pate et al. (19) point out that it is an important task for schools to promote health behavior because of the increasing number of overweight children and adolescents.

Previous school-based programs in primary schools in Germany showed only a limited impact on children's health and health behavior. The URMEL-ICE study found no short-term effect on BMI but on waist circumference and subscapular skinfold thickness (20). The program “Join the Healthy Boat” resulted in a short-term reduction in cardiovascular disease risk and SBM, improvements in motor skills and a tendency of higher physical activity pattern (21–23). Graf et al. (24) conducted the CHILT Project which aimed to promote a healthy lifestyle in primary schools and consisted of health education lessons, physical activity breaks, and games for leisure. After 4 years, they observed higher improvements in lateral jumping and balancing backwards in the intervention group (25). Concerning the measured endpoints, Dobbins et al. (17) conclude in their systematic review that school-based interventions to promote physical activity and fitness have little or no effect on television viewing behavior, physical activity, blood pressure, and BMI. In addition, the authors suggest that significant changes in physical activity were observed in programs with longer duration and that interventions with significant effects included changes in school curricula and printed teaching materials. The recent review by Love et al. (26) also concludes that school-based intervention programs do not lead to a change in physical activity and that there are no differences between girls and boys and between children from different socioeconomic backgrounds. Changing health and health behavior through school-based programs is a necessary task from the point of view of public health, but also a challenging one in order to achieve the required effects in the short and long-term.

Of particular note is the fact that a long-term evaluation of such projects and interventions over the years is rather rare: only 2 out of 23 school-based intervention studies that measured the impact on physical activity duration had a follow-up of 12 months or more (17).

In 2006, the German Heart Foundation initiated the *Skipping Hearts* project, which aims to promoting physical activity and an active lifestyle through rope skipping during childhood. Skipping rope is the modern form of jumping rope and strengthens physical fitness, complex movement patterns, and social skills in girls and boys (27). The project is free of charge for schools and contains a two-staged concept: a one-time 90-min Basic-Workshop (supervision of a trainer of the German Heart Foundation) and the subsequent Champion-Program with 10 45-min rope skipping lessons (standardized curricula, implementation by the teacher). All participating schools and classes receive a package of materials, including a teacher's handbook. Up until August 2017, 12,487 school classes had completed the Basic-Workshop and 2,924 school classes had received the Champion-Program. In total, more than 500,000 children participated in *Skipping Hearts*. Therefore, *Skipping Hearts* is one of the largest projects promoting physical activity in Germany. It is well-described in a short-term evaluation with a 5-month follow-up, where the positive effects of both project parts on physical activity, body composition, and motor skills were determined (28).

This study aimed to investigate the long-term effect of the established *Skipping Hearts* project on anthropometric and cardiovascular parameters, motor performance, self-assessed physical fitness level, physical activity, SBM, and HRQoL in children. Therefore, an additional follow-up examination 3 years later was conducted. The effect sizes of the short-term evaluation were small and it was therefore of interest whether the Basic-Workshop and Champion-Program led to the effects being maintained or strengthened in the long term or whether the effects disappeared. We hypothesized that only the Champion-Program leads to long-term improvements in the mentioned parameters and that the Basic Workshop shows no long-term effects.

## Materials and Methods

### Study Design

*Skipping Hearts* was evaluated in a non-randomized controlled longitudinal design between 2011 and 2016. The study included a fitness test (anthropometrics, health parameters, and motor skills), a health behavior questionnaire, and an accelerometer-based assessment of children (baseline: 10/2011-03/2012, follow-up: 03/2015-07/2015). Moreover, children's exercise capacity and HRQoL were evaluated in cross-section with a self-assessment and external assessment (parents). Informed consent was obtained from all children and parents.

### Sampling Procedures

The sample consisted of the same subjects as the short-term evaluation study—pupils in the 3rd and 4th grade of primary school in Upper Bavaria (28). Children who only received the 90-min Basic-Workshop comprised group SH-B. Group SH-CH consisted of pupils who received the Champion-Program in addition to the Basic-Workshop. The schools registered with the German Heart Foundation for the *Skipping Hearts* project. It was up to the schools to decide whether a class would be involved in the Basic-Workshop only (42 classes) or also in the Champion-Program (26 classes). The control group (18 classes) consisted of children from schools that had neither the Basic-Workshop nor the Champion-Program. Between baseline and follow-up, the former primary school children moved to secondary school and were invited individually to join in the follow-up examination at a sports hall near their former primary school. Of 1,662 children with baseline data (838 SH-B, 480 SH-CH, 344 control group), 1,124 children (67.62%) were invited during the recruiting process and 538 (32.37%) were lost to follow-up. The main reason for loss to follow-up was inaccessibility at their secondary schools due to missing contact data.

An a priori power analysis with a small effect size (*f* = 0.1) and a power of 80% revealed a required total sample of *n* = 246 and thus *n* = 82 per group. A total of 486 students out of 1,124 (43.2%) joined in the follow-up examination. Of these, 286 only participated in the Skipping Hearts Basic-Workshop (SH-B), 140 additionally received the Skipping Hearts Champion-Program (SH-CH), and 78 served as controls. The required sample size from the power analysis was achieved in SH-B and SH-CH, in the control group it was only slightly below. A total of 307 parents of the 486 children with longitudinal data answered a questionnaire at follow-up.

Of 105 children with baseline data for short-term accelerometer-based assessment, 43 (41.0%) again took part in the accelerometer-based measurement at follow-up, 27 (25.7%) could not be contacted and 35 (33.3%) refused to participate in the long-term assessment. Seven of 43 subjects did not provide any data and 16 did not meet the inclusion criteria. Due to the small sample size of 20 subjects with baseline and follow-up data, the accelerometer-based assessment was supplemented by a cross-sectional survey at follow-up: 6 children with non-usable baseline data and 102 children without prior activity measurement, who volunteered for accelerometer measurement, were included. In total, of 151 subjects with follow-up examination, 90 completed the activity measurement, 41 did not provide any data, and 20 did not meet the inclusion criteria.

Follow-up examination was voluntary, and the fitness test took place in the afternoon during the children's leisure time. For renewed participation in the study, all children got a gym bag and took part in a lottery game with 1 × 2 concert tickets or 1 × 2 tickets for a German professional soccer league match as the main prize.

### Materials

Blood pressure was measured using Mobil-O-Graph ambulatory blood pressure monitoring system (IEM Healthcare, Stolberg, Germany). After a rest period of at least 5 minutes, the measurement was taken on the left upper arm in a sitting position with the blood pressure cuff at heart level. Individual cuff size was determined by upper arm circumference (29). Based on German reference data, BMI was transformed into standard deviation scores (SDS), according to Kromeyer-Hauschild et al. (30) and systolic (SBP) and diastolic blood pressure (DBP) according to Neuhauser et al. (31).

Body fat was measured using the near-infrared technique (Futrex Advanced Body Fat Analyzer 6100 A/ZL, Futrex, Inc, Maryland, USA) on the right and left upper arm in the middle of the biceps brachii muscle (32). This method has high reliability and can be used in pediatric longitudinal studies to monitor changes in body fat over time (33). The mean of both measurements was used for further analyses.

Standing long jump, jumping sideways, 20-m sprint, and 6-min run were performed to conduct motor performance (34). The 6-min run was only performed in the follow-up examination. The measurement procedure is described in Postler et al. (28) and Graf et al. (24). SDS of these motor tests were calculated according to Bös et al. (34). To make a statement about general motor performance, a motor score was calculated from the three tests that were performed at all times (standing long jump, jumping sideways and 20-m sprint). This was composed of the mean value of the SDS values of the measured parameters and thus forms a single, meaningful parameter for motor performance. The test-retest reliability of the individual tests used ranges from α = 0.89 to α = 0.92 and the tests show a good overall validity (34). In our study, the reliability of the motor score was α = 0.71 for the baseline and α = 0.77 for the follow-up measurement.

Activity levels were recorded using the MoMo activity questionnaire (35). The physical activity score was calculated according to Prochaska et al. (36) as the mean value of the questions “How many of the last 7 days have you been physically active for at least 60 min a day?” and “How many days in a normal week are you physically active for at least 60 min a day?” The physical activity score has acceptable reliability and validity in comparison to accelerometer measurements (36). The Cronbach's alpha of the physical activity score was α = 0.76 for the baseline and α = 0.84 for the follow-up measurement. To assess SBM, children were asked about their daily consumption of TV/video, computer/Internet, gaming consoles, and mobile phone according to Lampert et al. (37) and estimation of their fitness level (How well do you think your physical performance is [=fitness]?) was collected in five categories from “very well” to “not at all well.” An SBM index (h/day) was built according to Finne et al. (16). Although the SBM index was used in the reported study, it has not yet been checked for reliability and validity. The reliability of SBM in our study was α = 0.49 for the baseline and α = 0.51 for the follow-up measurement.

A subsample performed an accelerometer-based measurement using Actigraph GT3X+ to assess objective physical activity, particularly the time spent in MVPA. Study participants should wear the accelerometer for 1 week all day on the right hip. Inclusion criteria were wearing the accelerometer at least 7 h daily on at least 3 workdays and 1 weekend day (38, 39). The cut-points of Freedson et al. (40) were used to determine activity intensity levels. An activity of fewer than 100 counts/min was generally defined as sedentary activity (41). Epoch length was 60 s. Data were classified as wear time and non-wear time according to the algorithm of Choi et al. (42). Besides, children recorded their daily physical activity and wear time in an activity protocol.

The KINDL-R questionnaire was used to assess the HRQoL of children through self-report and parent-report (43). The questionnaire was only used in the follow-up examination. It contains 24 items in 6 subscales (physical well-being, emotional well-being, self-esteem, family, friends, and school), whereby four items make up one subscale. Subscale scores and the total score are transformed from 0 to 100 with a higher score expressing a better HRQoL. In addition to HRQoL, parents were asked about their child's physical activity according to Prochaska et al. (36) (no personal data available). The psychometric characteristics of the KINDL-R revealed a reliability of α = 0.54 to α = 0.73 for the subscales and α = 0.82 for the total score (44). Satisfactory convergent validity is generally attributed to the subscales and the total score (45). The reliability of the scores in our study ranged from α = 0.43 (friends) to α = 0.74 (family) in the self-report and from α = 0.66 (friends) to α = 0.83 (physical well-being) in the parent-report. The overall HRQoL score reliability was α = 0.83 and α = 0.87 in the self-report and the parent-report, respectively.

Standard operation procedure for follow-up was identical to the standard operation procedure for baseline examination. All measurements were performed by previously trained staff.

### Statistical Analysis

Mean and standard deviations are reported for continuous data, median for non-normally distributed continuous data. Absolute and relative frequencies are shown for categorical data.

A comparison of baseline data of originally included participants who were willing to take part in the long-term assessments and those who refused to perform (children who could not be contacted were not considered). Welch two-sample *t*-tests were used to examine differences between children with follow-up and children without follow-up in anthropometrics, cardiovascular parameters, motor performance, physical activity, and SBM at baseline. Fisher's exact test was used for the categorical variable physical fitness in this analysis.

One-way analysis of variance for metric variables and Kruskal–Wallis tests for categorical variables were used for group comparisons at baseline.

Analysis of variance for repeated measures considering time as within-subjects variable and group as between-subjects variable for anthropometrics, cardiovascular parameters, motor performance, physical activity score, and SBM were used to examine changes between baseline and follow-up in total and between SH-B, SH-CH, and the control group over time for all 486 participants with baseline and follow-up data. For the categorical variable physical fitness, we used Wilcoxon signed rank test and Pearson's Chi-squared test in this analysis.

Due to the small sample size in the accelerometer-based assessment, Wilcoxon signed rank test was used to analyze the change between follow-up and baseline data in accelerometer data in total. We further calculated the differences between follow-up and baseline data and used Kruskal Wallis test to analyze the changes between groups, respectively.

Changes over time within groups were examined using paired *t*-tests for anthropometrics, cardiovascular parameters, motor performance, physical activity score, and SBM, and Wilcoxon signed rank tests for accelerometer data and assessment of physical fitness (Bonferroni correction *p* < 0.0167).

One-way analysis of variance was used to analyze only cross-sectional data of follow-up measurement: accelerometer (*n* = 90), 6-min run (*n* = 477), physical activity score (parental report), and HRQoL for both parental report (*n* = 307) and self-report (*n* = 455).

Statistical data analysis was performed using SPSS Statistics 23.0 (IBM Corp., Armonk, NY, USA) and R 3.3.3 (R Foundation for Statistical Computing, Vienna, Austria). The level of significance was set at *p* < 0.05 and *p* < 0.1 was set as the tendency of significance. Effect size is reported for all analyses of variance.

## Results

### Dropout-Analysis: Differences in Subjects With or Without Participation in Long-Term Assessment at Baseline

Children who participated in follow-up had better baseline values in BMI (16.7 ± 2.36 vs. 17.5 ± 2.99, *p* < 0.001), BMI-SDS (0.07 ± 0.96 vs. 0.20 ± 1.07, *p* < 0.001), body fat (15.3 ± 3.14 vs. 15.7 ± 3.63, *p* = 0.040), SBP (113.5 ± 10.9 vs. 115.1 ± 12.0, *p* = 0.022), SBP-SDS (1.34 ± 1.28 vs. 1.52 ± 1.37, *p* = 0.027) and motor performance (0.34 ± 0.73 vs. 0.08 ± 0.72, *p* < 0.001) than children who didn't participate in the follow-up (Table 1). No significant differences were found in the number of days/week with daily at least 60 min of physical activity (4.06 ± 1.82 vs. 3.87 ± 1.82, *p* = 0.108) and relative (22.5 ± 3.73 vs. 22.8 ± 6.61, *p* = 0.837), absolute minutes in MVPA per day (170.3 ± 30.3 vs. 168.2 ± 51.0, *p* = 0.866) and self-assessment of own physical fitness at baseline. Children with participation in long-term assessment showed a tendency of lower SBM (1.79 ± 1.99 vs. 2.09 ± 2.74, *p* = 0.075) at baseline.

**Table 1:** Dropout-analysis: differences in subjects with or without participation in long-term assessment at baseline.

	**Participation in long-term assessment**			**No participation in long-term assessment**			***p-*value**
**Welch two-sample *t*-tests**	***n***	**Mean (SD)**	**95% CI**	***n***	**Mean (SD)**	**95% CI**	
Age (years)	486	8.98 (0.60)	[8.93, 9.04]	638	9.09 (0.65)	[9.04, 9.14]	**0.005**
Height (cm)	478	135.1 (6.71)	[134.5, 135.7]	631	135.6 (6.87)	[135.0, 136.1]	0.260
Weight (kg)	478	30.7 (6.32)	[30.1, 31.3]	631	32.5 (7.78)	[31.8, 33.1]	**<0.001**
BMI (kg/m^2^)	478	16.7 (2.36)	[16.5, 16.9]	631	17.5 (2.99)	[17.3, 17.7]	**<0.001**
BMI-SDS	478	0.07 (0.96)	[−0.15, 0.02]	631	0.20 (1.07)	[0.11, 0.28]	**<0.001**
Body fat (%)	476	15.3 (3.14)	[15.0, 15.6]	626	15.7 (3.63)	[15.4, 16.0]	**0.040**
SBP (mmHg)	460	113.5 (10.9)	[112.5, 114.4]	618	115.1 (12.0)	[114.1, 116.0]	**0.022**
SBP-SDS	460	1.34 (1.28)	[1.22, 1.46]	618	1.52 (1.37)	[1.42, 1.63]	**0.027**
DBP (mmHg)	460	70.3 (9.08)	[69.4, 71.1]	618	71.0 (9.61)	[70.3, 71.8]	0.191
DBP-SDS	460	1.18 (1.39)	[1.06, 1.31]	614	1.28 (1.46)	[1.17, 1.40]	0.268
Motor performance	464	0.34 (0.73)	[0.28, 0.41]	608	0.08 (0.72)	[0.03, 0.14]	**<0.001**
Physical activity score (days/week)	405	4.06 (1.82)	[3.88, 4.24]	567	3.87 (1.82)	[3.72, 4.02]	0.108
MVPA relative (% of wear time)	20	22.5 (3.73)	[20.7, 24.23]	59	22.8 (6.61)	[21.1, 24.5]	0.837
MVPA absolute (minutes/day)	20	170.3 (30.3)	[156.1, 184.4]	59	168.2 (51.0)	[154.9, 181.5]	0.866
SBM (h/day)	365	1.79 (1.99)	[1.58, 1.99]	496	2.09 (2.74)	[1.85, 2.33]	0.075
**Fisher's exact test**		***n*** **(*****%*****)**			***n*** **(*****%*****)**		***p*****-value**
Physical fitness		454 (100)			614 (100)		0.169
Very well		197 (43.4)			230 (37.5)		
Well		191 (42.1)			271 (44.1)		
Medium		61 (13.4)			105 (17.1)		
Not as well		3 (0.7)			7 (1.1)		
Not at all well		2 (0.4)			1 (0.2)		

*Significant results marked in bold types. BMI, body mass index; SBP, systolic blood pressure; DBP, diastolic blood pressure; SDS, standard deviation score; MVPA, moderate-to-vigorous physical activity; SBM, screen-based media use*.

### Baseline Characteristics

Four hundred and eighty six children (256 female) had baseline and follow-up data with a mean age at baseline of 8.98 ± 0.60 years (Table 2). 10.0% were classified as underweight, 9.6% as overweight, and 2.5% as obese. Mean age in SH-B (9.06 ± 0.64) was ~2 months higher than in SH-CH (8.89 ± 0.59, *p* = 0.019) and in the control group (8.87 ± 0.46, *p* = 0.038). SH-B (4.13 ± 1.77, *p* = 0.033) and SH-CH (4.20 ± 1.87, *p* = 0.038) reported higher physical activity compared to controls (3.53 ± 1.82). Groups did not significantly differ in BMI, body fat, blood pressure, motor performance, objectively measured physical activity, and SBM (*p*-values not shown). At baseline, 85.4% of the participating children (SH-B: 87.9%, SH-CH: 83.6%, control group: 80.6%*, p* = 0.582) reported a good or very good own physical fitness.

**Table 2:** Descriptive statistics of baseline and follow-up data.

	**Baseline**		**Follow-Up**		***p*-value**	**η^**2**^**
**Paired *t*-tests from ANOVA for repeated measures**	**Mean (SD)**	**95% CI**	**Mean (SD)**	**95% CI**		
Age (years)	8.98 (.60)	[8.93, 9.04]	12.4 (0.60)	[12.3, 12.4]	**<0.001**	1.00
Height (cm)	135.1 (6.71)	[134.5, 135.7]	156.3 (8.22)	[155.6, 157.0]	**<0.001**	0.996
Weight (kg)	30.7 (6.32)	[30.7, 31.3]	45.6 (9.91)	[44.8, 46.5]	**<0.001**	0.873
BMI (kg/m^2^)	16.7 (2.36)	[16.5, 16.9]	18.5 (2.95)	[18.3, 18.8]	**<0.001**	0.557
BMI-SDS	−0.07 (0.96)	[−0.15, 0.02]	−0.13 (1.07)	[−0.22, −0.03]	**0.025**	0.010
Body fat (%)	15.3 (3.14)	[15.0, 15.6]	21.70 (5.34)	[21.2, 22.2]	**<0.001**	0.739
SBP (mmHg)	113.4 (10.8)	[112.2, 114.4]	119.2 (12.0)	[118.0, 120.3]	**<0.001**	0.153
SBP-SDS	1.34 (1.27)	[1.22, 1.46]	1.10 (1.26)	[0.98, 1.22]	**0.014**	0.013
DBP (mmHg)	70.2 (9.04)	[69.4, 71.1]	74.5 (8.19)	[73.8, 75.3]	**<0.001**	0.136
DBP-SDS	1.18 (1.38)	[1.06, 1.31]	1.33 (1.21)	[1.21, 1.44]	**0.027**	0.011
Motor performance	0.35 (0.73)	[0.28, 0.42]	0.51 (0.74)	[0.45, 0.58]	**<0.001**	0.057
Physical activity score (days/week)	4.06 (1.82)	[3.88, 4.24]	4.64 (1.69)	[4.47, 4.81]	**<0.001**	0.064
SBM (h/day)	1.77 (1.92)	[1.56, 1.97]	3.38 (2.38)	[3.12, 3.63]	**<0.001**	0.242
**Wilcoxon signed rank tests**	**Median**		**Median**			
MVPA relative (% of wear time)	22.6		8.7		**<0.001**	
MVPA absolute (minutes/day)	170.8		74.6		**<0.001**	
**Wilcoxon signed rank tests**	***n*** **(*****%*****)**		***n*** **(*****%*****)**			
Physical fitness	439 (100)		439 (100)		**<0.001**	
Very well	190 (43.3)		75 (17.1)			
Well	185 (42.1)		227 (51.7)			
Medium	59 (13.4)		123 (28.0)			
Not as well	3 (0.07)		14 (3.2)			
Not at all well	2 (0.05)		0 (0.0)			

*Significant results marked in bold types. BMI, body mass index; SBP, systolic blood pressure; DBP, diastolic blood pressure; SDS, standard deviation score; MVPA, moderate-to-vigorous physical activity; SBM, screen-based media use*.

### Changes Between Baseline and Follow-Up in Total

Height (Δ 21.2 ± 3.53, *p* < 0.001), weight (Δ 15.0 ± 5.08 *p* < 0.001), BMI (Δ 1.86 ± 1.47, *p* < 0.001), body fat (Δ 6.41 ± 3.39, *p* < 0.001), SBP (Δ 5.74 ± 13.1, *p* < 0.001), DBP (Δ 4.27 ± 10.3, *p* < 0.001), DBP-SDS (Δ0.14 ± 1.57, *p* = 0.027) and motor performance (Δ0.16 ± 0.57, *p* < 0.001) increased from baseline to follow-up (Table 2). Age- and sex-independent BMI-SDS (Δ −0.06 ± 0.52, *p* = 0.025) and SBP-SDS (Δ −0.24 ± 1.48, *p* = 0.014) decreased over time and prevalence of overweight and obesity remained constant (χ^2^ = 3.98, *p* = 0.409). Subjective physical activity score (Δ0.58± 2.22, *p* < 0.001) and daily SBM (Δ 1.61 ± 2.56, *p* < 0.001) increased between baseline and follow-up. A decline of MVPA-minutes (*z* = −3.92, *p* < 0.001) and percentage of MVPA-minutes of daily wear time (*z* = −3.92, *p* < 0.001) was observed. The assessment of own physical fitness declined between baseline and follow-up (*z* = −10.0, *p* < 0.001).

### Changes Between Baseline and Follow-Up Within Groups

Height, weight, BMI, SBP, DBP, and SBM increased between baseline and follow-up in all groups (Table 3). BMI-SDS only decreased in SH-CH (Δ −0.11 ± 0.49, *p* = 0.006). SH-B (Δ −0.34 ± 1.52, *p* < 0.001) and not SH-CH decreased in SBP-SDS. DBP-SDS did not change over time within all groups. Motor performance significantly increased over time in both project groups (SH-B: Δ0.17± 0.57, SH-CH: Δ0.16 ± 0.54, *p* < 0.001 for both). Between baseline and follow-up, self-reported physical activity score increased in SH-B (Δ0.53 ± 2.20, *p* = 0.001) and the control group (Δ 1.02 ± 2.04, *p* < 0.001), but not in SH-CH. Absolute and relative MVPA (*z* = −2.52, *p* = 0.012) significantly decreased over time in SH-CH. Between baseline and follow-up, all groups significantly decreased in their assessment of own physical fitness (SH-B: *z* = −7.48, SH-CH: *z* = −5.26, control group: *z* = −4.09, *p* < 0.001 for all).

**Table 3:** Descriptive statistics of baseline and follow-up data (SH-B, SH-CH, control group) and intra-group development over time.

	**SH-B**						**SH-CH**						**Control group**					
		**Baseline**		**Follow-Up**		*p*-value^*^		**Baseline**		**Follow-Up**		*p*-value^*^		**Baseline**		**Follow-Up**		*p*-value^*^
**Paired *t*-tests**	***n***	**Mean (SD)**	**95*%* CI**	**Mean (SD)**	**95% CI**		***n***	**Mean (SD)**	**95*%* CI**	**Mean (SD)**	**95% CI**		***n***	**Mean (SD)**	**95*%* CI**	**Mean (SD)**	**95% CI**	
Age (years)	268	9.06 (0.64)	[8.99, 9.14]	12.4 (0.64)	[12.4, 12.5]	**<0.001**	140	8.89 (0.59)	[8.79, 8.99]	12.3 (0.58)	[12.2, 12.4]	**<0.001**	78	8.87 (0.46)	[8.77, 8.97]	12.2 (0.47)	[12.1, 12.3]	**<0.001**
Height (cm)	260	135.3 (6.56)	[134.5, 136.0]	156.5 (7.71)	[155.6, 157.4]	**<0.001**	140	135.3 (7.07)	[134.1, 136.4]	156.5 (8.98)	[155.0, 158.0]	**<0.001**	78	134.2 (6.55)	[132.7, 135.7]	155.1 (8.46)	[153.2, 157.0]	**<0.001**
Weight (kg)	260	30.5 (6.19)	[29.8, 31.3]	45.5 (9.16)	[44.4, 46.6]	**<0.001**	140	30.9 (6.04)	[29.9, 32.0]	45.9 (10.24)	[44.2, 47.7]	**<0.001**	78	30.7 (7.27)	[29.1, 32.3]	45.6 (11.7)	[43.0, 48.3]	**<0.001**
BMI (kg/m^2^)	260	16.6 (2.26)	[16.3, 16.8]	18.5 (2.75)	[18.1, 18.8]	**<0.001**	140	16.8 (2.23)	[16.4, 17.2]	18.6 (3.03)	[18.1, 19.1]	**<0.001**	78	16.9 (2.88)	[16.2, 17.5]	18.8 (3.46)	[18.0, 19.5]	**<0.001**
BMI-SDS	260	−0.13 (0.92)	[−0.24, −0.01]	−0.17 (1.04)	[−0.30, −0.04]	0.209	140	0.03 (0.92)	[−0.13, 0.18]	−0.09 (1.06)	[−0.26, 0.09]	**0.006**	78	−0.03 (1.14)	[−0.29, 0.22]	−0.06 (1.20)	[−0.33, 0.21]	0.687
Body fat (%)	258	15.4 (3.18)	[15.0, 15.8]	21.9 (5.13)	[21.2, 22.5]	**<0.001**	139	15.3 (3.16)	[14.8, 15.8]	21.7 (5.42)	[20.7, 22.6]	**<0.001**	78	14.8 (2.97)	[14.2, 15.5]	21.3 (5.88)	[19.9, 22.6]	**<0.001**
SBP (mmHg)	246	113.7 (10.7)	[112.3, 115.0]	118.6 (12.4)	[117.0, 120.1]	**<0.001**	134	112.7 (10.7)	[110.9, 114.5]	119.1 (11.2)	[117.2, 121.0]	**<0.001**	77	113.8 (11.4)	[111.2, 116.4]	121.1 (12.2)	[118.3, 123.9]	**<0.001**
SBP-SDS	246	1.35 (1.27)	[1.19, 1.51]	1.01 (1.30)	[0.85, 1.18]	**0.001**	134	1.26 (1.29)	[1.04, 1.48]	1.11 (1.17)	[0.91, 1.31]	0.196	77	1.44 (1.25)	[1.15, 1.72]	1.36 (1.24)	[1.08, 1.64]	0.662
DBP (mmHg)	246	70.5 (9.00)	[69.3, 71.6]	74.1 (7.99)	[73.1, 75.1]	**<0.001**	134	69.6 (9.06)	[68.1, 71.2]	74.6 (7.55)	[73.3, 75.8]	**<0.001**	77	70.6 (9.21)	[68.5, 72.7]	75.6 (9.76)	[73.4, 77.8]	**<0.001**
DBP-SDS	246	1.21 (1.38)	[1.03, 1.38]	1.26 (1.19)	[1.11, 1.41]	0.571	134	1.09 (1.38)	[0.86, 1.33]	1.34 (1.10)	[1.15, 1.52]	0.075	77	1.26 (1.39)	[0.94, 1.57]	1.51 (1.44)	[1.18, 1.83]	0.202
Motor performance	260	0.37 (0.74)	[0.28, 0.46]	0.53 (0.71)	[0.45, 0.62]	**<0.001**	136	0.30 (0.69)	[0.18, 0.42]	0.46 (0.79)	[0.32, 0.59]	**0.001**	60	0.39 (0.81)	[0.19, 0.60]	0.57 (0.74)	[0.38, 0.76]	0.040
Physical activity score (days/week)	207	4.13 (1.77)	[3.89, 4.37]	4.66 (1.64)	[4.44, 4.89]	**0.001**	113	4.20 (1.87)	[3.85, 4.55]	4.63 (1.78)	[4.30, 4.96]	0.050	58	3.53 (1.82)	[3.06, 4.01]	4.55 (1.57)	[4.14, 4.97]	**<0.001**
SBM (h/day)	182	1.77 (2.00)	[1.48, 2.06]	3.32 (2.14)	[3.01, 3.64]	**<0.001**	97	1.78 (2.07)	[1.37, 2.20]	3.54 (2.99)	[2.94, 4.14]	**<0.001**	56	1.73 (1.31)	[1.38, 2.08]	3.26 (1.88)	[2.75, 3.76]	**<0.001**
**Wilcoxon tests**		**Median**		**Median**				**Median**		**Median**				**Median**		**Median**		
MVPA relative (% of wear time)	5	22.9		7.80		0.043	8	21.9		8.90		**0.012**	7	22.6		7.90		0.018
MVPA absolute (minutes/day)	5	171.0		67.0		0.043	8	164.5		76.6		**0.012**	7	188.6		67.9		0.018
**Wilcoxon tests**		***n*** **(*****%*****)**		***n*** **(*****%*****)**				***n*** **(*****%*****)**		***n*** **(*****%*****)**				***n*** **(*****%*****)**		***n*** **(*****%*****)**		
Physical fitness	289					**<0.001**	128					**<0.001**	72					**<0.001**
Very well		103 (43.1)		37 (15.5)				57 (44.5)		25 (19.5)				30 (41.7)		13 (18.1)		
Well		107 (44.8)		130 (54.4)				50 (39.1)		62 (48.4)				28 (38.9)		35 (48.6)		
Medium		25 (10.5)		66 (27.6)				20 (15.6)		37 (28.9)				14 (19.4)		20 (27.8)		
Not as well		2 (0.8)		6 (2.5)				1 (0.8)		4 (3.1)				0 (0.0)		4 (5.6)		
Not at all well		2 (0.8)		0 (0.0)				0 (0.0)		0 (0.0)				0 (0.0)		0 (0.0)		
Not at all well																		

* *Corrected according to Bonferroni (significant when p < 0.0167). Significant results marked in bold types. SH-B, skipping hearts basic; SH-CH, skipping hearts champion; BMI, body mass index; SBP, systolic blood pressure; DBP, diastolic blood pressure; SDS, standard deviation score; MVPA, moderate-to-vigorous physical activity; SBM, screen-based media use*.

### Changes Between Baseline and Follow-Up Between Groups: Long-Term Effect of *Skipping Hearts*

Mean changes in all anthropometric and cardiovascular parameters over time did not differ between groups (Table 4). In subjective physical activity, no change over time was observed in SH-B and SH-CH compared to controls (*p* = 0.243). A tendency of lower decline of MVPA-minutes in SH-B and SH-CH compared to controls (χ^2^ = 5.09, *p* = 0.078) but not in percentage of MVPA-minutes of daily wear time (χ^2^ = 2.92, *p* = 0.232) was found. Mean changes in SBM over time did not differ between groups. Self-reported physical fitness showed no interaction between group and time (χ^2^ = 0.30, *p* = 0.990).

**Table 4:** Δ (follow-up—baseline) and group/time-interaction effect in anthropometric and cardiovascular parameters, motor performance, physical activity score and screen-based media use.

	**SH-B**			**SH-CH**			**Control group**				**η^**2**^**
		**Δ** **(follow-up—baseline)**			**Δ** **(follow-up—baseline)**			**Δ** **(follow-up—baseline)**		***p*-value**	
**ANOVA for repeated measures**	***n***	**Mean (SD)**	**95% CI**	***n***	**Mean (SD)**	**95% CI**	***n***	**Mean (SD)**	**95% CI**	time*goup	
Age (years)	268	3.37 (0.04)	[3.37, 3.38]	140	3.37 (0.04)	[3.36, 3.37]	78	3.36 (0.07)	[3.35, 3.38]	0.100	0.009
Height (cm)	260	21.2 (3.39)	[20.8, 21.6]	140	21.3 (3.76)	[20.7, 21.9]	78	20.9 (3.60)	[20.1, 21.8]	0.791	0.001
Weight (kg)	260	14.9 (4.71)	[14.4, 15.5]	140	15.0 (5.44)	[14.1, 15.9]	78	15.0 (5.63)	[13.7, 16.2]	0.990	<0.001
BMI (kg/m^2^)	260	1.88 (1.40)	[1.71, 2.05]	140	1.80 (1.59)	[1.54, 2.07]	78	1.88 (1.49)	[1.54, 2.21]	0.873	0.001
BMI-SDS	260	−0.04 (0.53)	[−0.11, 0.02]	140	−0.11 (0.49)	[−0.20, −0.03]	78	−0.03 (0.55)	[−0.15, 0.10]	0.338	0.005
Body fat (%)	258	6.43 (3.24)	[6.03, 6.83]	139	6.37 (3.33)	[5.81, 6.93]	78	6.43 (3.99)	[5.53, 7.33]	0.986	<0.001
SBP (mmHg)	246	4.89 (13.5)	[3.20, 6.59]	134	6.41 (12.0)	[4.36, 8.46]	77	7.26 (13.5)	[4.19, 10.3]	0.299	0.005
SBP-SDS	246	−0.34 (1.52)	[−0.53, −0.15]	134	−0.15 (1.37)	[−0.39, 0.08]	77	−0.08 (1.52)	[−0.42, 0.27]	0.281	0.006
DBP (mmHg)	246	3.66 (10.0)	[2.40, 4.92]	134	4.92 (10.3)	[3.15, 6.68]	77	5.05 (11.1)	[2.54, 7.57]	0.399	0.004
DBP-SDS	246	0.06 (1.53)	[−0.14, 0.25]	134	0.24 (1.57)	[−0.02, 0.51]	77	0.25 (1.69)	[−0.14, 0.63]	0.438	0.004
Motor performance	260	0.17 (0.57)	[0.10, 0.24]	136	0.16 (0.54)	[0.07, 0.25]	60	0.17 (0.64)	[0.01, 0.34]	0.976	<0.001
Physical activity score (days/week)	207	0.53 (2.20)	[0.23, 0.83]	113	0.43 (2.33)	[0.00, 0.87]	58	1.02 (2.04)	[0.48, 1.55]	0.243	0.008
SBM (h/day)	182	1.55 (2.36)	[1.21, 1.90]	97	1.78 (3.10)	[1.21, 1.90]	56	1.53 (2.12)	[0.96, 2.09]	0.789	0.001

*SH-B, skipping hearts basic; SH-CH, skipping hearts champion; BMI, body mass index; SBP, systolic blood pressure; DBP, diastolic blood pressure; SDS, standard deviation score; SBM, screen-based media use*.

### Additional Analyzes at Follow-Up

Due to the little number of subjects with accelerometer data at both times of measurement, additionally data derived at the long-term assessment were analyzed without consideration of baseline data to estimate possible outlasting effects of SH. Groups did not differ in the daily MVPA-minutes (*p* = 0.568), percentage of MVPA-minutes of daily wear time (*p* = 0.488) and endurance capacity (*p* = 0.098, Table 5). In parental report, no significant group differences were determined in the physical activity score of the children (SH-B: 4.07 ± 1.73, SH-CH: 4.23 ± 1.74, control group: 3.91 ± 1.61, *p* = 0.605, η^2^ = 0.003). In self-assessment of children and parent-report, no significant group differences in total score and all subscales of KINDL-R were observed.

**Table 5:** Group comparison in 6-min run, MVPA, and HRQoL at long-term assessment.

	**SH-B**			**SH-CH**			**Control group**			***p*-value**	**η^**2**^**
**One-way ANOVA**	***n***	**Mean (SD)**	**95% CI**	***n***	**Mean (SD)**	**95% CI**	***n***	**Mean (SD)**	**95% CI**		
6-min run (meter)	262	1,076.6 (120.1)	[1,062.0, 1,091.2]	137	1,075.7 (122.5)	[1,055.0, 1,096.4]	78	1,043.8 (124.8)	[1,015.7, 1,072.0]	0.098	0.010
MVPA relative (% of wear time)	35	9.07 (3.70)	[7.80, 10.34]	27	9.99 (3.92)	[8.44, 11.5]	28	10.2 (4.03)	[8.58, 11.7]	0.488	0.016
MVPA absolute (minutes/day)	35	74.1 (29.9)	[63.8, 84.3]	27	80.8 (29.4)	[69.1, 92.4]	28	81.3 (31.6)	[69.0, 93.6]	0.568	0.013
KINDL-R self-report											
Total	261	74.0 (11.1)	[72.6, 75.3]	131	74.4 (12.2)	[72.3, 76.5]	63	73.7 (12.1)	[70.6, 76.7]	0.911	<0.001
Physical well-being	261	75.7 (16.9)	[73.6, 77.2]	130	75.4 (18.8)	[72.1, 78.6]	63	77.4 (17.1)	[73.1, 81.7]	0.739	0.001
Emotional well-being	259	82.0 (14.6)	[80.1, 83.7]	129	83.1 (14.9)	[80.6, 85.7]	62	82.3 (12.8)	[79.0, 85.5]	0.725	0.001
Self-esteem	259	55.7 (20.5)	[53.2, 58.2]	128	56.2 (22.8)	[52.2, 60.2]	61	56.0 (20.4)	[50.8, 61.2]	0.971	<0.001
Family	261	85.7 (16.3)	[83.7, 87.7]	131	84.8 (16.1)	[82.0, 87.6]	63	83.1 (17.3)	[78.8, 87.5]	0.534	0.003
Friends	258	78.0 (14.7)	[76.2, 79.8]	131	78.6 (15.9)	[75.8, 81.3]	63	76.1 (16.8)	[71.8, 80.3]	0.555	0.003
School	261	66.7 (17.9)	[64.5, 68.9]	131	68.0 (19.3)	[64.7, 71.4]	63	67.0 (20.2)	[62.0, 72.1]	0.807	0.001
KINDL-R parent-report
Total	177	78.6 (8.7)	[77.3, 79.9]	88	76.7 (10.2)	[74.5, 78.8]	40	75.4 (11.2)	[71.8, 79.0]	0.082	0.016
Physical well-being	177	81.3 (14.9)	[79.1, 83.6]	88	77.3 (17.9)	[73.5, 81.1]	40	75.9 (16.2)	[73.5, 81.1]	0.050	0.020
Emotional well-being	177	84.0 (11.1)	[82.3, 85.6]	87	82.1 (12.7)	[79.4, 84.8]	40	81.4 (14.1)	[76.9, 85.9]	0.303	0.008
Self-esteem	177	70.9 (13.8)	[68.9, 73.0]	88	69.5 (13.5)	[66.7, 72.4]	40	67.2 (14.8)	[62.5, 71.9]	0.288	0.008
Family	177	79.5 (15.0)	[77.3, 81.7]	89	79.4 (14.2)	[76.4, 82.4]	40	79.2 (12.0)	[75.4, 83.1]	0.991	<0.001
Friends	177	77.6 (12.7)	[75.7, 79.5]	88	76.5 (13.9)	[73.5, 79.4]	40	73.4 (15.3)	[68.6, 78.3]	0.210	0.010
School	175	78.3 (14.3)	[76.2, 80.5]	87	75.3 (17.2)	[71.6, 78.9]	40	75.2 (14.1)	[70.7, 79.7]	0.219	0.010

*SH-B, skipping hearts basic; SH-CH, skipping hearts champion; MVPA, moderate-to-vigorous physical activity*.

## Discussion

This study aimed to evaluate the long-term effects of the *Skipping Hearts* health promotion project, a donation-funded project of the German Heart Foundation. A positive effect of the *Skipping Hearts* Basic-Workshop and Champion-Program regarding the long-term improvement of health and health behavior could not be determined, but a tendency towards a less pronounced decrease of physical activity was observed.

While Postler et al. (28) found significant short-term improvements in motor performance and body fat (SH-B and SH-CH compared to controls) as well as activity level (SH-CH compared to controls), the long-term evaluation could not confirm the positive short-term results. SH-B and SH-CH did not show diverging development in anthropometrics, cardiovascular parameters, motor performance, SBM, and HRQoL compared to the control group between baseline and follow-up. Despite the tendency for *Skipping Hearts* to prevent an age-related decrease of physical activity, this result might be influenced by the high dropout rate of 44.9% in accelerometer measurement.

A decline in BMI-SDS and SBP-SDS was found in all groups. This indicates an age- and sex-independent improvement in these parameters. Furthermore, children in all groups improved their motor performance and physical activity score but the results of objective accelerometer data do not confirm this improvement. The decline within the 3.5 years from baseline to follow-up is in line with the literature: Physical activity decreases between childhood and adolescence (46–49). Even though a direct effect of *Skipping Hearts* was not found, participation in a study-related fitness test can be a motivational factor for children to increase their health status and fitness level. However, comparison of baseline data between children willing to participate in the long-term assessment and those who refused revealed that the healthier children participated again at follow-up. This indicates that the follow-up examination was not attended by those children who were the main target group of the project. Therefore, the current study population was more likely to stay in their initial healthy state.

A review of the relevant literature reveals that there are no consistent findings regarding the long-term effectiveness of prevention and health promotion projects at school. After a 1-year school-based physical activity intervention and a follow-up of 3 years, Meyer et al. (50) found a long-term increase in aerobic fitness compared to controls, but no sustainable effects on physical activity, cardiovascular parameter, and quality of life. Kobel et al. (22) also reported no significant increase in physical activity but a significant decrease in SBM in girls in a 1-year follow-up of the cluster-randomized longitudinal study “Join the Healthy Boat.” Anderson et al. (51) conducted a school-based cluster-randomized controlled trial including lessons-plans, teaching materials, parental-child homework activities, training for teachers, and health promotion strategies for parents; but they observed no effect on MVPA and sedentary behavior. In contrast, Lahti et al. (52) reported higher levels of physical activity compared to controls 4 years after ending a 7-year intervention at school. Furthermore, Vander Ploeg et al. (53) observed a greater increase in activity on school days and on weekends in the intervention group 2 years after the implementation of a comprehensive school health program.

In a Cochrane Review of 2013, Dobbins et al. (17) suggest that school-based interventions only have limited effects regarding BMI, blood pressure, pulse rate, and physical activity level. This is interesting insofar as only one out of 44 had a follow-up measurement at 4 years after intervention and the majority of studies had the point of measurement directly post-intervention. Because the findings of every new intervention study are consistent with those from other studies, the probability of improving the tested parameters in a short-term intervention study is high. However, statements regarding the effectiveness of an intervention should be treated with caution because they do not reveal anything about a long-term effect. A long-lasting increase in activity levels and motor performance should be the main goal of every project related to prevention and health promotion research. Therefore, it is not surprising that *Skipping Hearts* is not able to sustainably enable children to do more physical activity. Indeed, the short-term effectiveness of *Skipping Hearts*—both the Basic-Workshop and the Champion-Program—has to be viewed as an unexpected success.

*Skipping Hearts* is not a controlled and scientifically supported intervention, but a donation-funded project of a foundation. The Champion-Program of *Skipping Hearts* is planned as a continuous implementation of 10 45-min prepared lessons. The responsibility of implementation lies within the school administration because there is no supervisor. To a certain extent, the implementation of the Champion-Program is therefore entirely up to the teachers themselves. It may well be that this individual responsibility for the implementation process is the most crucial point for its effectiveness. It can be assumed that teachers implemented the lessons during and not in addition to sports education. On measurement days at school, teachers gave this verbal feedback and mentioned that they did not meet the given guidelines—the large majority did not conduct all 10 lessons and reduced the physical activity duration. To get a detailed insight into the assessment of the project and its conception and implementation into the daily routine of schools, qualitative interviews were conducted with the teachers and headmasters of 64 participating schools. Furthermore, a nationwide survey of all teachers and headmasters (*n* = 1474) who participated in *Skipping Hearts* since its beginning in 2006 was performed. These data are currently being analyzed and will be published elsewhere.

To achieve a long-term effect of the Champion-Program, it may be therefore necessary to instruct trained supervisors at school who serve as contact persons for all teachers. The fact that this concept can be successful is shown by Vander Ploeg et al. (53) in the evaluation of a comprehensive school health approach. A key component of the intervention was the placement of a full-time school health facilitator at each school, who was dedicated to promoting healthy living. Furthermore, Lahti et al. (52) postulate that education for a sustainable active way of living is possible when interventions are implemented in the daily routine of schools. It therefore may be necessary to strive for sustainable implementation of rope skipping in the daily routine of schools. Offering lessons in shorter bouts must not necessarily harm the effectiveness of the Champion-Program. In their systematic review, Barr-Anderson et al. (54) highlight that short, 10–15-min, bouts daily can increase physical activity—if these bouts are implemented in the daily routine of schools.

Taken these findings together, several points impair the proposed project goal, specifically the sustainable motivation of children to be physically active all their lives. Therefore, we recommend considering the following suggestions in the future program planning of *Skipping Hearts*: The Champion-Program should be conducted according to the guidelines of the German Heart Foundation and should be implemented in the daily routine of schools after the end of the project to obtain sustained rope skipping. The project should also force the transfer of knowledge about health behavior, and all involved teachers should undergo specific training. Furthermore, a supervisor should be provided for all schools, who serves as a contact person if problems occur while implementing the project. Teachers should directly motivate pupils to practice rope skipping as part of their school routine (e.g., during their school break) and should integrate parents further into the project (e.g., practicing together at home). Nevertheless, the easy and uncomplicated character of the *Skipping Hearts* project should remain unchanged. With rope skipping, the project offers a physical activity that can be performed in children's everyday life without high costs.

The results are limited due to the non-randomized design of the study, specified by Postler et al. (28). *Skipping Hearts* is not a scientifically supported and controlled intervention but a school project that was carried out under everyday conditions. In addition, the long-term evaluation of *Skipping Hearts* was added subsequently to the short-term evaluation. Therefore, the recruitment of the “original” sample turned out to be very time-consuming and difficult. Since the previous evaluation was not designed for the long-term, there were no contact details for the participants. Children could no longer be reached via the former elementary schools, as they had meanwhile moved on to a secondary school. Therefore, intensive research was carried out in all secondary schools in the catchment area of the former primary schools, and a variety of measures were undertaken to provide information about the continuation of this short-term evaluation. Nevertheless, the missing data could have biased the results. Given that the follow-up examination was voluntary and took place in the afternoon, compounded with the problem that many children could not even be invited due to missing personal data, the participation rate of 43.3% is quite successful. Furthermore, baseline differences between children with and without follow-up of the anthropometric parameter and motor skills could have led to biased results. It should be noted that children answered the questionnaire on their own without the control of teachers or parents. Restrictions regarding the project itself are described in Postler et al. (28). Although good reliability is reported for near-infrared technology in children and adolescents, body fat measured with near-infrared technology is overestimated compared to densitometry by hydrostatic weighing (33). However, the same measurement methodology was applied at all measurement points, as the NIR is suitable for longitudinal studies. The socio-economic status of the parents, the migration background, and the nutritional behavior of the children were not sufficiently investigated in this study. These confounding factors could influence both the collected parameters and the effectiveness of *Skipping Hearts*, which we did not record within this study.

In summary, the observed short-term effects of *Skipping Hearts* could not be confirmed after 3 years. We did not observe long-term effects on children's cardiovascular health, motor skills, physical activity, or quality of life. Nevertheless, *Skipping Hearts* can certainly be used as an initial impulse, but for a long-term effect, the project needs to be revised and schools should be more proactive regarding the promotion of students' health. In addition, every program promoting physical activity and motor skills in youth should strive for the long-term, and intervention studies should test a sustainable effect.

## Data Availability Statement

The datasets analyzed within the study are available from the corresponding author on reasonable request.

## Ethics Statement

The studies involving human participants were reviewed and approved by German Sport University Cologne (project number 113/2014). Written informed consent to participate in this study was provided by the participants' legal guardian/next of kin.

## Author Contributions

TP, CG, NF, RO-F, and TS designed the study. LB, TP, and TS performed the data collection. BH performed the data analysis. LB and TP assisted in data analysis and drafted the manuscript. All authors reviewed, edited, and approved the manuscript.

## Conflict of Interest

The authors declare that the research was conducted in the absence of any commercial or financial relationships that could be construed as a potential conflict of interest.

## Abbreviations

BMI: body mass index
DBP: diastolic blood pressure
HRQoL: health-related quality of life
MVPA: moderate-to-vigorous physical activity
SBM: screen-based media use
SBP: systolic blood pressure
SDS: standard deviation score
SH-B: skipping hearts basic group
SH-CH: skipping hearts champion group.
